# The Examination for Sugar in the Urine

**Published:** 1860-03

**Authors:** Ch. Leconte

**Affiliations:** Paris


*The Examination for Sugar in the Urine.* By Ch. Leconte, of Paris.

The author examines the action of various tests alleged to be satisfactory in the way of determining the presence of sugar in the urine. He enumerates Trommer's test, ammoniated copper, cuprotartrate of potassa, solution of potassa, lime-water, solution of chromic acid acidulated with chlorhydric or sulphuric acid, and the simultaneous employment of sub-nitrate of bismuth and solution of caustic potassa. The use of these reagents should never induce one to conclude, *absolutely*, as to the presence or absence of sugar in the urine, as he proceeds to show; although they can render real service when sugar exists in a somewhat notable quantity in the urine.

1. *Trommer's Method.*—This consists in adding to the urine a small quantity of solution of sulphate of copper, then an excess of potassa, and finally to heat the liquid to ebullition, depending on the property sugar possesses, in oxydizing, of depriving the oxide of copper of one-half of its oxygen, and thus reducing it to the condition of *red* oxide, insoluble in the solution of potassa—a result which is very easily accomplished. Nothing is more simple than the application of this process, with the understanding, always, that an excess of sulphate of copper be not employed, for the excess of the oxide present, on which the sugar could not act, would be converted by the temperature into its anhydrous form, and its *black* color would mask the *red* color of the suboxide.

2. *The Cuprotartrate of Potassa* does not present the same objection as that just named, but when it is prepared according to Barreswil's directions, and kept on hand for some time, it often happens that a red precipitate is afforded when it is boiled alone, or after an addition of equal or double volumes of water.

3. *Fehling's Liquor* differs from that of Barreswil, in the substitution of soda for potassa, and can be kept on hand for a longer time; but, as I have determined a number of times, in numerous fruitless experiments, which I have made to find a liquid test for sugar free from reproach, Fehling's liquor is less sensitive than Trommer's method, or that of Barreswil, and it often does not indicate the presence of half a thousandth of sugar added to urine.

4. *Glycerine* has been proposed as a substitute for tartaric acid in the copper solution. The suggestion, although very ingenious, has given me results but little satisfactory, since this liquid, at the end of a few days, deposits in the cold a considerable quantity of the red oxide of copper, and almost invariably, if boiled immediately after its preparation, will exhibit reddish flakes, showing the beginning of the process of reduction.

These four liquids present this inconvenience, that they can be reduced by a *large number* of substances, and particularly by uric acid, as I have satisfactorily demonstrated. Furthermore, even when sugar or uric acid is present, they may be decolorized instead of furnishing a red precipitate, if ammoniacal salts or urea are present in proper quantity. One can very readily be satisfied of this by experiment with the latter reagents when pure.

In order to avoid the trouble from the presence of urea, the author, in his experiments on the urine of females during lactation, adopted the following plan:

"Four litres (about a gallon) of the urine, which quite rapidly reduced the cupro-potassa liquid and reddened litmus paper, were acidulated with acetic acid and evaporated over a water-bath in porcelain capsules; the evaporation was rapid, on account of the shallowness of the vessels. When about eight-tenths of the urine had evaporated, it was allowed to cool, and then alcohol of 38° was gradually added, so as to precipitate most of the mineral salts, and so as to have an alcoholic liquid sufficiently dilute to retain the sugar in solution. This liquid was evaporated to dryness; the residuum drenched with alcohol at 40°, which dissolved out the urea, and left undissolved the sugar and some mineral salts soluble in dilute alcohol. Such a dilute alcohol solution would enable us to employ Trommer's test with some show of accuracy."

5. The other processes for the detection of glucose are: the brown coloration which solutions of potassa, soda, baryta, strontia or lime, or even of ammonia, assume in the presence of glucose—this characteristic is worth nothing when search is made for sugar in the midst of a large number of substances whose action on alkalies has not been studied, since the question becomes, then, Is glucose the only substance which imparts a brown color to urine under the action of alkalies? No one, at present, can answer in the affirmative. The coloring substances of the urine are hardly known, and many substances will assume a brown color under the influence of alkalies, which is heightened by heat. All the extractive substances are in this category.

6. The brown color, assumed by nitrate of bismuth, under the influence of potassa in the presence of urine, cannot be considered a peculiar action of sugar, as other reducing agents will produce the same effect.

In conclusion, we see that these secondary actions or characteristics may furnish some useful information as to the presence of glucose in the urine, without being endowed with certainty, since they are also produced by other substances; they have no real value except when united to the essential characteristics.

*Essential characteristics* are such as belong *alone* to glucose, and are the production of the alcoholic fermentation, and the extraction of the glucose itself.

*Alcoholic Fermentation.*—Notwithstanding the interesting investigations that have been published, in modern times, on this subject, yet it remains a fact, that cane sugar and glucose are the only substances that, in contact with yeast, undergo regular fermentation, furnishing pure carbonic acid and alcohol. Any liquid which does not evolve carbonic acid, after two hours' contact with yeast and exposure to a temperature between 68° and 86°, should be considered as free from sugar, unless, however, the volume of the gas formed be much less than that of the liquid containing the sugar. Indeed, yeast should only be *directly* added to urine in cases of very decided diabetes; when it is necessary to search for sugar in urine containing but a small quantity, it is proper to concentrate the sugar after the method already described, and then to expose the substance which has been isolated with a small quantity of water and yeast to a temperature from 68° to 86°; fermentation will then take place rapidly. In order to be satisfied that the gas discharged does not proceed from any alteration of the yeast, a small quantity of the latter should be exposed along with pure water in a tube alongside of that in which the test has been applied.

The best arrangement for the accurate employment of the fermentation test is this: a large test-tube is taken, closed at one end; to the other end a cork is adapted, through which is passed a tube of small bore, drawn out very narrow at the lower end, which should reach almost to the bottom of the large tube; the upper end of the tube should pass through the cork about an inch, and remain open. The test-tube is filled with the mixture of urine and yeast, then the cork with the smaller tube is introduced—a small quantity of the liquid will run out through the open end of the small tube. The large tube is then placed in lukewarm water—fermentation taking place, the gas rises to where the cork is inserted, and pressing downward, gradually forces out the liquid, which escapes from the upper extremity of the small tube. In order to test the nature of the gas, the open extremity of the small tube is plunged into a solution of potassa; the closed end of the test-tube is heated by a spirit-lamp, so that a few bubbles of gas may escape; the lamp being removed, the gas cools, contracts; the solution of potassa enters, filling it entirely, if aided by slight agitation.

*Extraction of the Sugar.*—In the present condition of science, we feel authorized to declare, that sugar is present when fermentation yields carbonic acid and alcohol; yet, strictly speaking, the separation of the sugar itself only should permit us to give a positive opinion as to its presence. Now, the process for the extraction of sugar is so delicate that we could obtain it, although there were only five centigrammes in 200 grammes of urine. Leconte's process is as follows, being a modification of Lehmann's: The urine is slightly acidulated with sulphuric acid, since the mineral sulphates are all insoluble in alcohol; then it is evaporated in very shallow dishes until a pasty residuum is obtained, to which is added, in the warm, a small quantity of alcohol at 33°, in order to dilute it. This is placed in a flask and exposed to a boiling heat; the alcohol poured off, and more added, so that the residuum shall be exposed to the action of boiling alcohol two or three times. The liquids thus obtained are poured into one vessel, heated and filtered; after they have cooled, a saturated alcoholic solution of caustic potassa, recently prepared, is added in small quantities, strong agitation being used after each addition; the liquid, which at first becomes turbid, clears up by the deposition of a pasty substance on the sides of the flask. The potassa is added so long as it produces any turbidness; then the clear liquid is decanted, and the deposit in the flask is removed by rinsing it carefully with alcohol. This deposit is then dissolved in a small quantity of water; its potassa is precipitated by excess of tartaric acid and agitation, and the bitartrate of potassa is removed by filtration. The acid liquid, in the cold, is treated with chalk in excess—being agitated from time to time, until it becomes perfectly neutral to test-paper, when it is refiltered, evaporated in a water-bath, and the residuum treated with alcohol. This alcoholic liquid, being allowed to evaporate spontaneously, furnishes a syrup, which, after a long time, (one of my specimens required eight months,) gives small, four-sided, prismatic crystals, with diedral summits.

When, instead of extracting the sugar, it is only desired to try the fermentation test, it will answer to saturate the aqueous solution of the precipitate, produced by the potassa, with dilute sulphuric acid; the sulphate of potassa, being but slightly soluble, subsides, especially when agitation is used; it is then separated by the filter, and the filtrate, being diluted with a small quantity of water, is mixed with yeast, and placed in the fermentation apparatus.

The precipitate referred to in the preceding paragraph is mostly urate of potassa, when it will not undergo the process of fermentation. This can thus be proven: after the precipitate is dissolved in a small quantity of water, if the solution be heated with a slight excess of acetic acid, and then allowed to remain in a cool place for a few hours, on treating it with alcohol, crystals of uric acid will separate, which can be recognized by the microscope, or by their transformation into murexide under the action of nitric acid.—*Gazette Médicale, Oct.* 8, 1859. L. H. S.

